# Intraoperative Neuromonitoring of the External Branch of the Superior Laryngeal Nerve during Thyroidectomy: The Need for Evidence-Based Data and Perioperative Technical/Technological Standardization

**DOI:** 10.1155/2014/692365

**Published:** 2014-11-24

**Authors:** Alberto Mangano, Georgios D. Lianos, Luigi Boni, Hoon Yub Kim, Dimitrios H. Roukos, Gianlorenzo Dionigi

**Affiliations:** ^1^1st Department of General Surgery Ospedale di Circolo e Fondazione Macchi Varese, Department of Human Morphology and Surgical Sciences Endocrine Surgery Research Center, Insubria University Varese-Como, 21100 Varese, Italy; ^2^Department of General Surgery, Ioannina University Hospital and Centre for Biosystems and Genomic Network Medicine Ioannina University, 45110 Ioannina, Greece; ^3^Department of Surgery, KUMC Thyroid Center, Korea University Anam Hospital, Korea University College of Medicine, Seoul, Republic of Korea

## Abstract

The external branch of the superior laryngeal nerve (EBSLN) is surgically relevant since its close anatomical proximity to the superior thyroid vessels. There is heterogeneity in the EBSLN anatomy and EBSLN damage produces changes in voice that are very heterogenous and difficult to diagnose. The reported prevalence of EBSLN injury widely ranges. EBSLN iatrogenic injury is considered the most commonly underestimated complication in endocrine surgery because vocal assessment underestimates such event and laryngoscopic postsurgical evaluation does not show standardized findings. In order to decrease the risk for EBSLN injury, multiple surgical approaches have been described so far. IONM provides multiple advantages in the EBSLN surgical approach. In this review, we discuss the current state of the art of the monitored approach to the EBSLN. In particular, we summarize, providing our additional remarks, the most relevant aspects of the standardized technique brilliantly described by the INMSG (International Neuromonitoring Study Group). In conclusion, in our opinion, there is currently the need for more prospective randomized trials investigating the electrophysiological and pathological aspects of the EBSLN for a better understanding of the role of IONM in the EBSLN surgery.

## 1. Surgical Anatomy and EBSLN Classification

The superior laryngeal nerve (SLN) is a branch of the vagus after its exit from the skull base. It usually originates at the nodose ganglion close to the jugular foramen at the level of C2 (about 4 cm cranially to the carotid artery bifurcation) and it descends posterior to the carotid arteries toward the larynx [1–3]. During this course, the SLN crosses posterior to the internal carotid artery and anterior to the superior cervical sympathetic ganglion/cervical sympathetic chain [2]. En route, the SLN descends in a medial direction toward the thyrohyoid membrane and at a variable distance from its origin (0–20 mm, average 15 mm); usually approximately at the hyoid bone superior cornu [2], the superior laryngeal nerve (SLN) divides into its branches: the internal and external one [1].

The EBSLN derives, in the upper region of the neck, from the SLN and typically crosses the internal carotid artery (75%) or the common carotid artery posteriorly and descends medial to the middle cervical sympathetic ganglion and posterior to the superior thyroid artery (STA). En route, the cricothyroid artery (a STA branch) joins to the nerve supplying it for most of its course [2]. Lateral to the thyroid cartilage, the EBSLN lies more rarely in the pretracheal fascia or more commonly in the visceral fascia [2]. As a result of the attachment of the sternothyroid muscle to the thyroid cartilage (its oblique line), the EBSLN and the superior thyroid artery and vein (having their course deep to the sternothyroid muscle) have a parallel route beneath the muscle [2].

In summary, the EBSLN has its course dorsal to the carotid sheath and then crosses in a medial direction towards the larynx. En route, the EBSLN is dorsal to the superior thyroid artery and in a superficial location to the inferior pharyngeal constrictor muscle as it goes caudally and travels in a medial direction to innervate, on the lower portion of the cricoid cartilage, the cricothyroid muscle [1, 2]. Moosman and DeWeese described the sternothyroid-laryngeal triangle as the area where the EBSLN approaches the larynx: this region is defined anteriorly by the sternothyroid muscular fibers, laterally by the superior thyroid pole (laterally retracted), and medially by the inferior pharyngeal constrictor muscle and cricothyroid muscle [1, 4]. In the aforementioned triangular area the EBSLN is typically in a medial location, the superior thyroid vein is laterally located, and the superior thyroid artery is usually between the nerve and the vein. In some cases, the EBSLN, of note, can be in adherence (15%) to the superior thyroid artery.

Normally, the nerve is situated adjacent to the inferior pharyngeal constrictor and is not enclosed by the capsule of the thyroid gland [2]. After the EBSLN has its course in a caudal direction laterally to the larynx on the inferior pharyngeal constrictor muscle, at the cricoid level the EBSLN usually divides into two branches, with a separate entrance at the pars obliqua and pars recta of the cricothyroid muscle bellies [1]. The EBSLN total length (unaffected by sex, ethnicity, and the side of the neck) ranges between 31.5 mm and more than 90 mm [2] and it is about from 0.2 to 0.8 mm wide [1, 2]. Sañudo et al. found that in almost 70% of cases, the EBSLN after the innervation of the cricothyroid muscle extends through the cricothyroid membrane to innervate the anterior thyroarytenoid muscle region [5]. Moreover, some findings about the existence of cross-innervation by the EBSLN of the thyroarytenoid muscle has been shown [6, 7]. Additionally, according to Maranillo et al. [8] there is in 85% of cases a neural connection between the external laryngeal nerve and the RLN and the human communicating nerve. Hence, the human communicating nerve gives documented connection to the vocal fold from 41% to 85% of cases [1, 5–8]. This neural connection variability is clinically relevant, because during EBSLN IONM, not in all cases, there are some recordable waveforms. This inconstancy monitoring finding is probably explained by the aforementioned variability in neural connection, alongside the variable EBSLN stimulation-related wave-form, and variability in endotracheal tube position [1].

The EBSLN is surgically relevant because it is in close anatomical proximity to the superior thyroid vessels. In most cases, the EBSLN extends in a medial direction to the larynx, hence cranial to the superior thyroid pole, and should be therefore be at minimal risk for iatrogenic damage during the approach of the superior pole. Unfortunately, there is heterogeneity in the caudal neural extent (see classification systems discussed below) and in large goiter or short neck, the surgical risk is considerable [1, 2, 9]. Notably, the* de visu* EBSLN detection is impossible in almost 20% of cases because of a neural location deep to the inferior constrictor muscle fascia [10, 11]. With IONM, even those nerves that are subfascial and not directly visualizable should be theoretically monitored [11].

Notably, the most commonly used EBSLN surgical classification has been proposed by Cernea et al. [9]: in 23% of large goiters and 68% of small goiters, the EBSLN crosses the superior thyroid vessels more than 1 cm above the superior thyroid pole (Type 1). Moreover, in 15% of large goiters and 18% of small goiters, the EBSLN crosses the vessels less than 1 cm above the superior pole (Type 2A). Finally, in 54% of large goiters and 14% of small goiters, the EBSLN crosses the superior thyroid pedicle below the upper border of the superior thyroid pole (Type 2B) [9]. Because of their low-lying course, Types 2A and 2B are more prone to iatrogenic injury [5, 9]. Moreover, Kierner et al. [3], published another classification: the authors introduced a fourth EBSLN type (more difficult to* de visu* identify and present in 13% of cases), having its course dorsal to the superior thyroid vessels [3].

What is more, more recently some other interesting (but at this stage less frequently used) EBSLN classification systems have been proposed by Selvan et al. [12] and Friedman et al. [13]. All those aforementioned anatomical considerations are very relevant because the complete anatomical knowledge of this region (in particular to precisely evaluate the cricothyroid muscle twitch) is the conditio sine qua non and the prerequisite for an accurate surgical technique [1].

## 2. Neurophysiological and Neuropathological Aspects

During its course the SLN (when it crosses posterior to the internal carotid artery and anterior to the superior cervical sympathetic ganglion/cervical sympathetic chain) receives a sympathetic branch from the superior cervical sympathetic ganglion. This branch contributes to the innervation of the thyroid gland [14] and the carotid body [15]. This anatomic loop has been well described by several authors and more than 20 variations have been described so far [2]. Sun and Chang [14] and Furlan [16] showed that, in up to almost 80% of the cases, the sympathetic chain is mainly in connection to the EBSLN [2].

What is more, the EBSLN motor fibers (the ones important in terms of IONM) are directed to the cricothyroid muscle (composed by two segments: the pars obliqua and the pars recta), whose action is adjusting the vocal fold tension and length [1, 17]. The vocal fold thickness and tension (which are related to the vocalis portion of the thyroarytenoid muscle and cricothyroid muscle) are responsible for the voice timbre (which in turn is linked to the vocal fold vibration frequency).

EBSLN damage produces changes in voice projection and quality [18] alongside alterations in the high-pitched sounds production ability [19]. However, the clinical consequences of such event are very variable and heterogeneous and can be very subtle and difficult to assess/diagnose: ranging from a hoarse or weak voice to no clinical finding at all [1, 20]. Those symptoms may be, of note, more noticeable with professional speakers (above all among women) [21].

## 3. Incidence, Diagnosis, and Management of EBSLN Injury

The real prevalence of EBSLN injury is hard to evaluate because currently there are too many intertrial methodological differences. In fact, the prevalence of such event widely ranges from near 0% to approximately 60% [1, 22–27].

EBSLN dysfunction related symptoms may, in some cases, deeply and negatively influence the standard quality of life [1, 28]. However, endoscopic postsurgical evaluation (in stark contrast to RLN injury) does not show constant standardized findings and clinical findings are not accurate and globally the real incidence of nerve impairment is underestimated (EBSLN iatrogenic injury is considered the most commonly underestimated complication in endocrine surgery) [1, 22–27].* De facto*, the vast majority of them are subclinical or subtle and widely heterogeneous in nature, debatable, not standardized, and not conclusive [1, 28]. Hence, cricothyroid muscle EMG is the most accurate tool to diagnose EBSLN dysfunction (technical details of such a procedure are described in the INMSG guidelines) [1, 28, 29].

Notably, if attention is paid during the dissection of the superior thyroid pole to preserve the EBSLN, the nerve damage rate is decreased. Additionally, over the last years the increased IONM application in surgical settings improved the neural detection rate and reduced the percentage of neural damage (for RLN and for EBSLN) [1, 28–31].

Cernea et al., in case of lack of intraoperative EBSLN identification, reported an incidence ranging from slightly more than 10% to almost 30% [9]. In the same way, Jansson et al., if there was not an intraoperative EBSLN identification, reported a temporary EBSLN damage rate near to 60% and a permanent injury rate near to 4% [22]. What is more, another trial demonstrated that even if there is not a neural visual detection, the distal ligation of the superior thyroid vessels seems to be a safe procedure if carried out by experienced hands. However, in this case the temporary EBSLN damage prevalence is superior to the nerve detection technique (0.5% versus 0.8%) [32].

After temporary EBSLN injury, in some cases, there can be vocal improvement; what is more, phoniatric exercises can increase residual muscle performance. Unfortunately, when the EBSLN injury is total and bilateral, currently there is no way to regain dynamic pitch range [1]. However, promising data has been shown by El-Kashlan et al.: these authors performed, in a limited sample with high vagal lesions, selective cricothyroid muscle reinnervation. After the restorative procedure, in all the patients electromyographic and voice quality implementation was present [33].

At the end of the surgical procedure, intraoperative EBSLN monitoring can provide postoperative neural function prognostication: in particular, after the EBSLN stimulation (performed in the most cranial EBSLN segment, i.e., cranially to the superior pole region surgically managed) a positive cricothyroid twitch is a reliable evidence for functional EBSLN preservation [1].

## 4. Standardized Surgical Monitored Technique

In order to decrease the risk for EBSLN injury, multiple surgical approaches have been described so far: a nerve stimulator or intraoperative neuromonitoring use for confirmation and neural mapping of the EBSLN detection [9, 29, 34]; moreover, the* de visu* neural detection before superior thyroid vessels sealing has been reported [35]; additionally the sealing of the superior vascular branches under vision on the thyroid capsule without visual EBSLN identification has been presented [32].

Notably, when a new technology/technique is presented, its standardization is essential for many reasons: for safety to keep the obtained standards and for interoperability, repeatability, and comparison of results in an evidence-based way and via a scientifically rigorous methodology [28]. In this way, the INMSG recently published the guidelines for the EBSLN monitoring where the standardized surgical approach is brilliantly described in detail [1].

At this stage, in our opinion those technical recommendations represent the cornerstone of the EBSLN monitored surgical approach, and besides being followed in clinical settings, they should be in particular used in future experimental studies to obtain intertrial standardized and comparable results. In this section, we summarize the most relevant aspects of that standardized technique additionally providing some remarks (see Section 5).

In order to preserve the cricothyroid muscle and the EBSLN, an accurate surgical technique is mandatory and a deep anatomical knowledge of the several EBSLN variations is essential. The EBSLN course must be* de visu* detected or monitored to avoid potential neural damage when the superior pole is surgically approached [1]. The space between the superior pole and the cricothyroid muscle is avascular [2]. In this region, a superior pole blunt dissection should be performed to create a clear operational control and exposure of the sternothyroid-laryngeal triangle (location of the EBSLN) [1]. In most cases, the transverse division of the strap muscles is not needed and a gentle laterocaudal traction of the lobe can guarantee a good exposure (however, if necessary a partial transection of the sternothyroid muscle may implement the surgical field) [1]. The superior thyroid vessels must be identified, and individual branches exposed [1]: the EBSLN is usually parallel to the superior thyroid artery and located on the inferior constrictor muscle (prior to its termination within the cricothyroid muscle) [2]. The visual neural search is important but impossible in almost 20% of the cases because EBSLNs are subfascial/intramuscular [13]. What is more, in many cases nonneural tissue can be misidentified for a neural structure [12].

According to the INMSG, in all patients, the EBSLN should be* de visu* identified (when possible) and neural stimulation via cricothyroid muscle (CTM) twitch assessment and glottis endotracheal EMG monitoring should be performed [1]. The superior thyroid pole dissection is more risky for the EBSLN if large goiters or short necks are present because of the increased adherence of the nerve to the gland [36]. Notably, if the thyroid is >100 g, Type 2B nerve (more prone to surgery-related damage) is more frequent (up to 54%) [9, 29]. Considering all the aforementioned reasons, since it is unsafe for the EBSLN, mass ligation technique should not be used [1].

What is more, if energy-based devices are misused, additional risk of neural iatrogenic thermal lateral spread injury is present [37]. In this way, IONM can be a valid adjunct to the visualization technique alone [28]. Moreover, another advantage of the monitored approach is when a suture ligation or clip is applied near the EBSLN: in this case if IONM suggests a neural dysfunction, the clip or the suture must be removed in order to avoid risk of permanent neural damage [1].

### 4.1. Technique A EBSLN IONM: Stimulation-CTM Twitch

The principles highlighted in detail in the International Standards Guideline Statement [38] for the RLN can be applied to the EBSLN stimulation and monitoring but some relevant differences must be considered [1]. According to the INMSG (International Neuromonitoring Study Group) the surgical management of the superior pole should include the EBSLN* de visu* detection (when possible) and the following two neural monitoring steps (that implement the neural preservation rate) should be obtained [1].

(1) In order to have a true positive stimulation (i.e., after the stimulation of the ipsilateral nerve with 1 mA, in the presence or absence of the corresponding EMG response, via the CTM twitch correct identification of the EBSLN is confirmed): the EBSLN must be tested cranially and medial to the vascular pedicle of the superior pole [1].

(2) In order to obtain a true negative (i.e., the CTM twitch is absent after the stimulation of the non-EBSLN tissue) stimulation it is mandatory to exclude the presence of neural tissue in the pedicle that is to be divided [1].

Through the aforementioned monitoring maneuvers, the EBSLN absence is demonstrated and the surgical dissection is safer [1]. Selvan et al. reported a 100% EBSLN identification rate via CTM EMG and nerve stimulation and, of note, many false positives have been reported when* de visu* detection alone was used prior to electrical stimulation [12]. Notably, Barczyński et al. [34] in a prospective randomized controlled study of visualization versus EBSLN IONM showed that EBSLN detection rate was superior via IONM (less than 35% without NIM versus almost 85% with NIM). IONM reduced the prevalence of transient but, of note, failed to reduce the permanent EBSLN damage rate [34]. What is more, Lifante et al. also found a greater rate of EBSLN intraoperative identification, 65% with neuromonitoring versus more than 30% without IONM [39]. Furthermore, Aina and Hisham to identify the EBSLN used a nerve stimulator in a sample of 200 nerves at risk: the EBSLN identification rate has been more than 95% in primary thyroid surgery but, of note, in redo cases has been 65.0% [40].

Additionally, Dionigi et al. reported that, even during video-assisted thyroidectomy, the EBSLN identification rate in the IONM-group was almost 85% compared to more than 40% in the non-IONM arm [41].

*De facto*, EBSLN visual detection without EMG confirmation can be in some cases misleading [1]. IONM has the benefit of detecting all nerve anatomic variations (including Cernea Types 2A and B and Type 1 which are most prone to iatrogenic injury) [34]. Hence, INMSG recommends IONM use (current set at 1 mA) to assess EBSLN presence/absence during superior pole dissection.* De visu* neural identification can be confirmed placing the probe directly on the nerve at the entry point into cricothyroid muscular fibers [1]. Structures to be assessed should be stimulated parallel and underneath the sternothyroid muscle (its laryngeal head inserts onto the thyroid cartilage lamina), which is a reliable milestone for EBSLN distal identification prior to its termination into the CTM [42]. Notably, if a neural mapping is performed (current amplitude is increased from 1 mA to 2 mA) a relevant advantage achievable only via the IONM is that the EBSLN can be definitively identified even when the nerve is not* de visu* detectable (i.e., up to 20% of the cases) [1].

Finally, the CTM twitch is the confirmation for the positive neural identification, which in some cases can be also coupled with an audio signal and an EMG monitoring signal. What is more, after the superior thyroid pole surgical approach, that positive neural signal is useful for the documentation of the neural functional integrity and for prediction of the EBSLN postoperative function. This technique is valid for open thyroidectomy and for MIVAT as well [1, 41].

### 4.2. Technique B EBSLN IONM: Stimulation-Glottic EMG

In stark contrast to RLN, EBSLN monitoring is based on the assessment of CTM twitch (present in all patients) and an electromyographic response (not present in all patients but in a percentage ranging from 70% to 80%). The reason why EBSLN results in detectable waveform only in some cases is still unclear, but it may be due to some equipment-related limitations. In fact, the generated waveform is small in amplitude and short in latency; hence it may not be detected by monitoring software [1]. Furthermore, this EBSLN EMG heterogeneity may be explained by the fact that the EBSLN (that innervates primarily the anterior one-third of the cord) measurement may be very sensitive to endotracheal tube positioning [1]. Moreover, Selvan et al. have shown that good waveforms with 0.5 mA are possible [12] and this heterogeneity could be additionally related to the nerve position, distance, or the small size of such fibers. Massive scientific and technological effort is currently in progress to develop new cutting-edge monitoring systems capable to detect in all cases EBSLN EMG signal.

Multiple and heterogeneous different nerve-monitoring systems have been studied, but for safety and simplicity reasons, endotracheal tube-based surface electrodes are the most common [1].* De facto*, those systems with a graphic monitoring documentation are preferred because of audio-only systems lack of EMG response stimulation assessment (threshold, amplitude, latency, and waveform shape) [1, 30]. On the other hand, stimulating electrodes may be monopolar or bipolar. The latter may theoretically provide less stimulation artifacts and potential greater sensitivity [1].

RLN and EBSLN have some common monitoring standards of equipment setup, endotracheal tube placement, anesthesia, and correct tube positioning verification tests [1]. According to the INMSG the stimulator probe should be set at values ranging from 1 to 2 mA and the event threshold at 100 microV [1]. In particular, for* de visu* detection EBSLN confirmation, a 1 mA current should be preferred but higher values (up to 2 mA) should be set for EBSLN mapping. If a bipolar device is used, the precise orientation of the anode and cathode stimulating electrodes placed on the nerve is of crucial importance. Moreover, the bipolar probe may not be the best option for nerve mapping since there is a more focal stimulation in comparison with the monopolar device, providing more diffuse current spread, and may facilitate the mapping of a wider region [1].

Barczyński et al. [34] recently showed that, after a 1 mA stimulation of the EBSLN, the mean amplitude (detection system via surface electrodes on the endotracheal tube) was present in almost 75% of the samples. Notably, the mean amplitude of evoked potential after RLN stimulation was more superior than EBSLN stimulation [34]. Similar findings were recently showed in an interesting paper by Potenza et al.: after EBSLN stimulation an EMG waveform was obtained in almost 80% of the samples and the mean amplitude of the EBSLN amplitude was approximately one-third of the RLN [43].

Since there is great heterogeneity in amplitude values (and influence of tube position on those values), we think that it is currently crucial to assess what is the normality range of EBSLN monitoring.

## 5. Discussion

A damage of the EBSLN is associated with cricothyroid muscle motility impairment, altering the high tones production ability, the voice and frequency. This nerve is surgically relevant because of its proximity to the superior thyroid vessels: for those reasons those vessels must be carefully approached in order to avoid iatrogenic injury [1, 28].* De facto*, in just 15% of the cases the EBSLN is protected from surgical manipulation via a location far from the superior pole vessels. Iatrogenic injuries can be avoided via an accurate anatomic localization during the surgical dissection [9, 44, 45].

Unfortunately, very often surgeons do not even try the identification of this nerve [28]. For the aforementioned reasons, there is an increasing trend to create new technologies for EBSLN localization. Magnifying glasses are also available to identify and preserve the laryngeal nerves, hence reducing the morbidity [46]. Notably, Berti et al. [47] reported a 65% EBSLN detection rate in video-assisted thyroidectomy with the aid of an optical magnification endoscope based visualization guidance. In this way, IONM has been proposed as an adjunct to the standard technique of intraoperative* de visu* detection of the laryngeal nerves. IONM additionally provides a reliable identification and neuromonitoring of the superior laryngeal nerves during neck surgery [12, 41, 48–50]. Notably, Dionigi et al. showed that the EBSLN was more often identified in the IONM arm than in a control group without IONM (83.6% versus 42%), [41].

At this stage, there is the need for a EBSLN monitoring and anatomic detection standardized technique. In particular, the IONM technique standardization is crucial for many aspects: for safety, to obtain better surgical outcomes, to keep the obtained standards, for educational purposes, and for interoperability, repeatability, and ratification [28, 51]. The intact neural function of the entire EBSLN must be assessed via a stimulation of the most superior and caudal neural region exposed. On the contrary, indirect EBSLN stimulation via vagal nerve assessment can be difficult to perform in routine surgical settings, since it requires more extensive surgical dissection; however it may be applicable when lateral compartment lymph node dissection is indicated [28].

The good timing for the assessment of the intact EBSLN function must be after thyroidectomy and hemostasis. Recently, Masuoka et al. [11] published an interesting prospective randomized trial enrolling patient who underwent thyroid surgery for carcinoma. The visual and electrostimulatory identification rate of the EBSLN was the primary endpoint and the changes in the postoperative voice performance were the secondary endpoint. In particular, 203 nerves at risk were enrolled in the monitoring arm (even if the monitoring was not performed via an endotracheal tube with surface electrodes) and 202 nerves at risk were in the conventional technique group using a simple nerve stimulator (Vari-Stim 3) to assess the CTM twitch. The authors concluded that neuromonitoring allowed an improved visualization rate (48.8% versus 17.8%) and implemented electrostimulatory detection rate (5 times superior to conventional technique, i.e., 89.2% versus 17.8%) in comparison to the conventional surgical approach. Additionally, in the NIM arm there was a patient complaining of subjective voice impairment reduction [11].

Srithan et al. recently presented [52] an innovative paper with the aim to assess the normative latency and amplitude of the RLN, vagus nerve, and EBSLN applying those to postsurgical neural function documentation. Those nerves showed individual heterogeneous and unique electrophysiologic characteristics that can be recorded as electrophysiological evidence of the existence of postresection neural function [52].

Furthermore, Darr et al. [53] showed that IONM, using the novel endotracheal tube (Trivantage EMG tube), can be safely used for EBSLN identification in 100% of patients and the EBSLN EMG activity was assessed in 100% of cases as well. Moreover, the standard monopolar stimulator and the novel bipolar stimulator devices produced comparable EMG data [53].

Stroboscopy is, of note, an important tool to evaluate if IONM reduces the EBSLN damage rate [12, 28, 39, 41].

In conclusion, for all the aforementioned elements, IONM can provide multiple potential advantages in the EBSLN surgical approach: clinically there is a better neural detection rate and monitoring, what is more it can provide additional advantages for legal, research, and educational aims [28].

*De facto*, the neural functional impairment of the EBSLN during surgery is usually a rare event; hence it is not easy to obtain an adequate statistical power. However, to perform well-powered studies obtaining statistically supported data is a mandatory step to elucidate the role of new technologies/techniques in terms of detecting differences among the heterogeneous surgical techniques [31].

In order to better standardize the normative neural electrophysiological values of EBSLN, at this stage, there is currently the urgent need for more in-depth trials investigating the electrophysiological and pathological aspects of the EBSLN. Hence, prospective randomized trials are needed for a better understanding of the role of IONM in the EBSLN surgery and for the standardization of the EBSLN IONM technique [28, 31].
